# Recognition of Chiral Carboxylates by Synthetic Receptors

**DOI:** 10.3390/molecules26216417

**Published:** 2021-10-24

**Authors:** Patryk Niedbała, Kajetan Dąbrowa, Sylwia Wasiłek, Janusz Jurczak

**Affiliations:** Institute of Organic Chemistry, Polish Academy of Sciences (PAS), 01-224 Warsaw, Poland; kdabrowa@icho.edu.pl (K.D.); wasilek.syl@gmail.com (S.W.)

**Keywords:** supramolecular chemistry, anion recognition, chiral recognition, carboxylates, synthetic receptors

## Abstract

Recognition of anionic species plays a fundamental role in many essential chemical, biological, and environmental processes. Numerous monographs and review papers on molecular recognition of anions by synthetic receptors reflect the continuing and growing interest in this area of supramolecular chemistry. However, despite the enormous progress made over the last 20 years in the design of these molecules, the design of receptors for chiral anions is much less developed. Chiral recognition is one of the most subtle types of selectivity, and it requires very precise spatial organization of the receptor framework. At the same time, this phenomenon commonly occurs in many processes present in nature, often being their fundamental step. For these reasons, research directed toward understanding the chiral anion recognition phenomenon may lead to the identification of structural patterns that enable increasingly efficient receptor design. In this review, we present the recent progress made in the area of synthetic receptors for biologically relevant chiral carboxylates.

## 1. Introduction

A large number of molecules found in living organisms are chiral, which consequently allows for interactions between them, and this is the domain of chiral recognition [1,2,3,4,5]. The most spectacular fact is that enantiomers of chiral molecules can induce completely different reactions in living organisms. This applies, for example, to the binding of small chiral molecules by macromolecules (protein receptors) in natural communication pathways (neurotransmitters, hormones), sensory perception (different smells of enantiomers), or the action of synthetic drugs (e.g., the discovery of the mutagenic character of the (*S*) enantiomer of thalidomide) [6]. Moreover, chiral recognition is also important for analytical chemistry during the interaction of the analyte with the stationary phase during chromatographic separation, extraction to the solid phase, or transport across membranes [7]. For the above reasons, it becomes particularly important to understand the basis of chiral recognition. Despite such great importance of the discussed issue, the problem of chiral recognition using synthetic receptors, in comparison with molecular recognition of achiral guests, is relatively poorly understood. Designing a receptor that effectively discriminates chiral anions involves knowledge of previous literature reports and computational modeling of receptor–guest complexes, together with the experience and chemical intuition of the researcher. Despite the current studies on the synthesis and evaluation of chiral receptors, *a priori* prediction of their enantio-differentiating properties before synthesis and without time-consuming and expensive studies is still impossible. This is due to the fact that even small changes in the structure of the receptor can have a non-negligible effect on its role in the enantio-differentiation of chiral molecules, making it difficult to define general principles for the design of selective receptors. As a consequence, there is a deficit of works devoted to fundamental studies determining the influence of structural factors on the enantioselectivity of model receptors and the durability of their formation of diastereomeric complexes.

New, precisely designed chiral receptors also open up a number of practical opportunities. One area that has been strongly explored by many research groups is asymmetric synthesis [8,9,10,11]. Despite many successes in this area, the practical derivation of homochiral compounds still remains a challenge. Research on chiral recognition may enable the design of efficient catalysts for asymmetric catalysis or create chiral stationary phases for the separation of racemic mixtures. Furthermore, chiral receptors can be used as ion carriers in transport processes or can provide membrane fragments as sensors for chiral ions.

**Figure 1:**
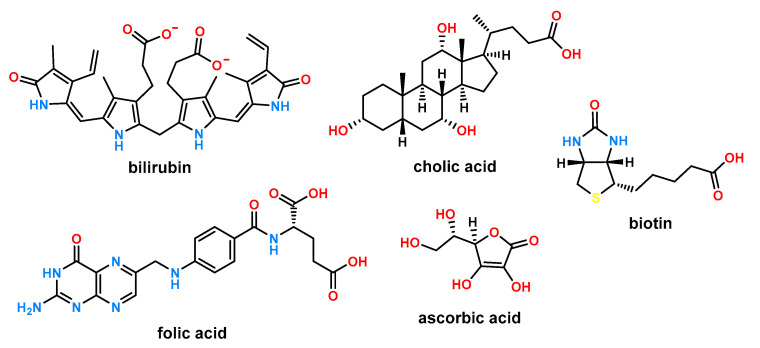
Examples of bioactive compounds having a carboxyl group.

A major challenge has been to design a host structure that will selectively interact with the carboxylate ion [12]. Carboxylates are examples of anions with a pseudotrigonal shape, and their negative charge is delocalized mainly on two oxygen atoms. Due to the presence of the carboxyl group in many bioactive compounds, including bilirubin, bile acids, folic acid (vitamin B9), ascorbic acid (vitamin C), and biotin (vitamin H) (Figure 1), in combinations with potential use in medicine, or in those taking an active part in many metabolic processes, carboxylates are essential objects in studies of anion association, if only because of their therapeutic importance. Moreover, a significant number of them are optically active compounds which stimulates the development of a chiral variant for their recognition.

The basis of chiral recognition is the formation of diastereomeric complexes due to the interaction of a chiral receptor with a chiral substrate. The chiral recognition phenomenon takes place when there is a difference in Gibbs free energy (ΔG) between the formed diastereomeric complexes. As previously mentioned, despite the many literature reports on this process, the multitude of factors affecting the chiral recognition, as well as the difficulty in predicting their contribution to the association process, makes the precise design of an effective receptor an extremely challenging task. Even small changes in any of the factors can have unanticipated and strong effects on selectivity and binding strength. It is still virtually impossible to predict the enantio-differentiating properties of a receptor before synthesis and time-consuming and expensive studies are conducted. As a result, there are few works with a fundamental approach to chiral recognition [13]; moreover, the lack of general rules for the selection, both of the solvent and the structure of the tested anions, makes it difficult to reliably compare ligands known from the literature with each other.

During a careful analysis of the literature reports, two main strategies emerge for the construction of the chiral anion receptors. The first concept assumes symmetric attachment of the anion-binding functions to a rigid platform matched to the carboxyl anions and then attachment of the chiral element. On the other hand, the second strategy takes advantage of the inherent chiral properties of the molecule, which after proper attachment of hydrogen bonding donors, allows formation of a chiral binding pocket. Prominent among the most commonly chosen chiral building blocks are readily available and inexpensive natural sources of chirality, such as amino acids [14,15,16], sugars [17,18], cyclodextrins [19,20], steroids [21], and synthetic sources—macrocycles or calixarenes [22]. These building blocks can be further modified to achieve the desired stereochemical properties. When talking about chirality, one has to take into account that molecules can also be characterized, in addition to the best known and most common center chirality, by axial chirality, e.g., atropoisomeric *ortho*-substituted biphenyls (BINOL derivatives) and allenes, or planar chirality, e.g., (*E*)-cyclooctene atropoisomerism or ferrocene derivatives. In this paper, we present a literature review on synthetic receptors for chiral carboxylates with a particular emphasis on the classification of the most commonly used hydrogen bonding donors.

## 2. Amide Receptors

**Figure 2:**
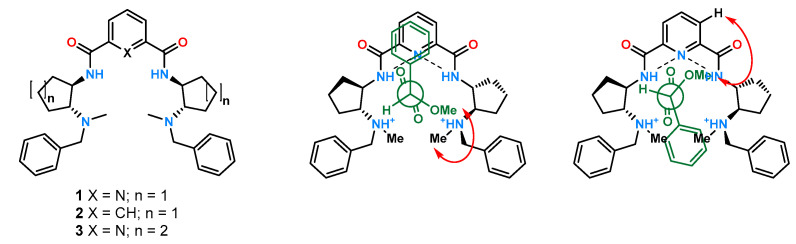
Chiral receptors **1**–**3** incorporating chiral 1,2-disubstituted cyclic amines.

Diamide derivatives of simple aromatic compounds have found significant use as platforms in chiral anion recognition. Taking their advantages, a family of 1,2-disubstituted cyclic amine derivatives **1**–**3** (Figure 2) was obtained and investigated [23,24].

The ligands were designed to compare the enantiodiscrimination abilities of the chiral 1,3-diamide derivative of phthalic acid containing a five-membered cyclopentane ring **2** with the analogous 2,6-disubstituted pyridine derivative **1** and the effect of alkane ring size for the derivatives **1** and **3**. The chiral recognition properties of receptors toward carboxylic acids were evaluated in CDCl_3_ using the ^1^H NMR technique by exploiting the ability of receptors to separate the reference protons in their racemic mixtures. Pyridine chiral solvating agents (CSAs) **1** and **3** resulted in a higher separation of the studied signals. Moreover, the ligand having a six-membered ring showed ΔΔδ ≥ 0.04 ppm for all tested guests. It was demonstrated that the tested receptors could be used as CSAs.

**Figure 3:**
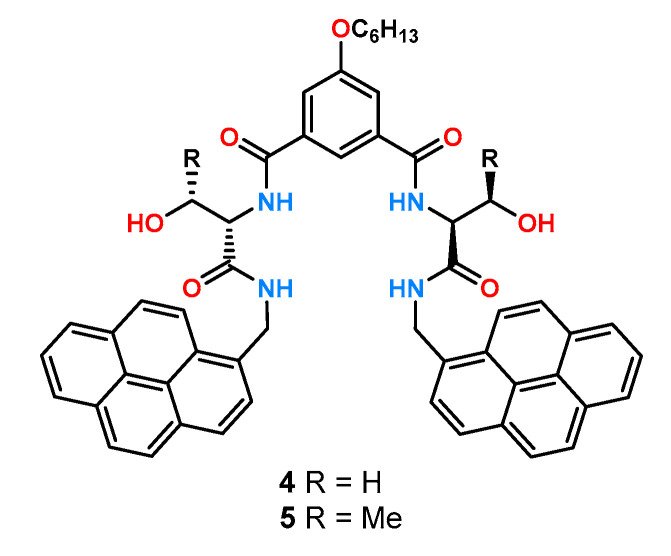
Tetraamide fluorescent receptors **4** (with serine) and **5** (with threonine).

In 2018, a paper was published on treating the chiral recognition of tetraamide fluorescent receptors **4** and **5** using the geometry of a diamide derivative of isophthalic acid (Figure 3) [25].

Symmetrically attached pyrene units were responsible for the fluorescence response, while the blocks introducing chirality were serine or threonine derivatives. Comparison of the binding properties and ability to enantioselectively complex anions measured by fluorescence changes in acetonitrile showed that **5**, having higher steric hindrance near the binding pocket, exhibited lower stability constants of the complexes formed (stability constants for the acetylated leucine derivative K*_D_* = $1.03\times 10^{5}\;M^{-1}$ and K*_L_* = $2.62\times 10^{4}\;M^{-1}$ and K*_D_* = $6.19\times 10^{4}\;M^{-1}$ and K*_L_* = $1.33\times 10^{4}\;M^{-1}$, for receptors **4** and **5**, respectively), while showing better chiral recognition (for leucine K*_D_*/K*_L_* = 3.9 and K*_D_*/K*_L_* = 4.6 for **4** and **5**, respectively). The obtained enantioselectivity for the threonine host **5** was confirmed by titration under ^1^H NMR control in DMSO-*d_6_*, where the calculated ratio of stability constants was K*_D_*/K*_L_* = 2.10 for leucine. The results obtained indicate a significant effect of the solvent medium on chiral recognition.

**Figure 4:**
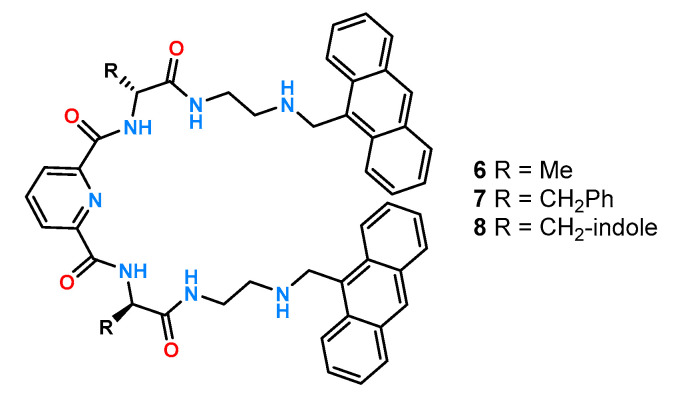
Receptors **6**–**8** based on 2,6-pyridinediamide motif decorated with **α**-amino acids and anthracene moieties.

A similar approach was used to construct the group of receptors **6**–**8** [26], where a diamide derivative of pyridine acted as the binding pocket, while the chiral block was made up of alanine, phenylalanine, or tryptophan chiral linkers (Figure 4).

Due to the attachment of anthracene units, it was possible to perform experiments by fluorescence titration in DMSO, while phenylalanine protected by a *tert*-butoxycarbonyl group (Boc) was taken as the reference anion. The obtained results presented in Table 1 show that the best enantioselective properties were exhibited by receptor **8** containing an indole derivative in its structure which allows the formation of additional hydrogen bonds.

**Figure 5:**
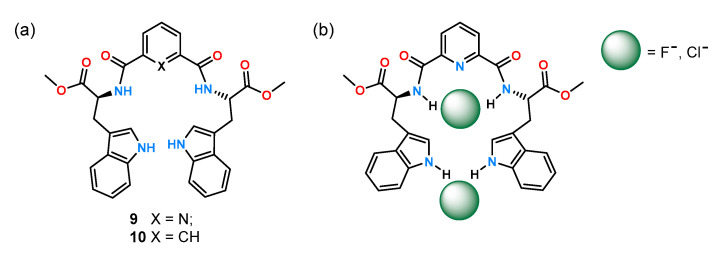
(**a**) Receptors **9** and **10** incorporating the tryptophan and indole moieties and (**b**) proposed 1:2 (receptor: anion) binding mode of receptor **9** with fluoride and chloride anions.

Previous work by the Gale group [27] allowed preparation of the symmetric receptors **9** and **10** (Figure 5) containing tryptophan moieties by the Caltagirone group [28]. The anion binding properties of receptors to both achiral and chiral anions were evaluated by ^1^H NMR titration experiments in DMSO-*d_6_* with 0.5% H_2_O.

While the isophthalic derivative **10** practically did not interact with the tested anions, receptor **9** formed complexes with stoichiometry 1:2 with chloride and fluoride anions and with an acetate complex with stoichiometry 1:1. Therefore, it was decided to continue the enantioselectivity studies only for receptor **9**. As it turned out, this host showed greater affinity for the *L* enantiomer of the serine and alanine derivatives tested, and the chiral recognition for them was at the level of K*_L_*/K*_D_* = 1.40 and K*_L_*/K*_D_* = 1.65, respectively.

**Figure 6:**
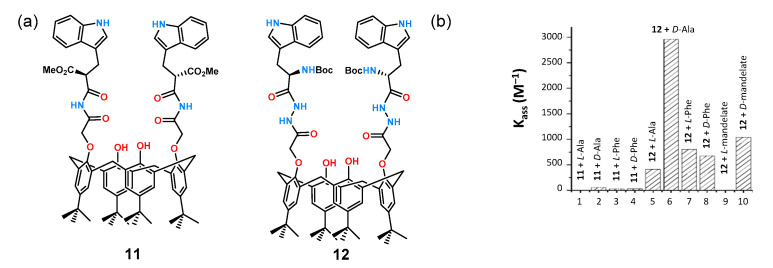
(**a**) Structures of receptors **11** and **12** and (**b**) their chiral recognition properties.

Tryptophan is commonly used as a chiral building block in the construction of receptors for anions because of its synthetic availability allowing straightforward preparation of a binding pocket around an inherently chiral molecule. A translation of this approach is shown in Figure 6a for receptors **11** and **12** based on the calixarene scaffold [29].

The receptors have two tryptophan molecules symmetrically attached via amide bonds to form an anion binding site. Affinity studies for a series of chiral carboxylate anions derived from mandelic acid, phenylglycine, *N*-Boc-phenylglycine, and *N*-Boc-phenylalanine were performed by fluorescence titration in DMSO. The titration results show the dependence of the stability constants (*K*_ass_) of the formed complexes of receptors **11** and **12** with the aforementioned anions (Figure 6b). As can be seen, receptor **11**, having only two amide functions, practically did not interact with the tested anions, while receptor **12** showed very good chiral recognition toward a pair of enantiomers of alanine and mandelic acid anions.

**Figure 7:**
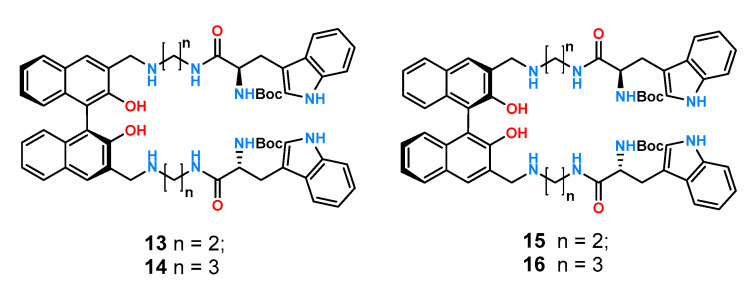
Receptors **13**–**16** based on BINOL decorated with tryptophane.

1,1′-Bi-2-naphthol (BINOL) is another structural motif important in the design of molecular receptors due to its inherent chirality, rigidity, and good synthetic availability. For these reasons, it was utilized in the construction of cation [30,31] and anion receptors [32,33] as well as in the preparation of chiral catalysts [34,35,36]. Taking advantage of the rigid structure of BINOL, four new ligands **13**–**16** (Figure 7) were prepared and studied by fluorimetric titration experiments in chloroform [37].

All receptors exhibited high association constants with the carboxylate anions tested which is understandable in such a non-competitive solvent. Furthermore, the highest chiral recognition was obtained for the pair of phenylalanine enantiomers protected by the Boc group K*_S_*/K*_R_* = 5.92 for **13** and K*_S_*/K*_R_* = 5.13 for **14**, with enantioselectivity reversals of K*_S_*/K*_R_* = 1/5.18 and K*_S_*/K*_R_* = 1/4.49 for **15** and **16** for *R*-BINOL, respectively.

## 3. Urea and Thiourea Receptors

The urea and thiourea groups are important anion binding motifs in the construction of molecular receptors for chiral anions.

**Figure 8:**
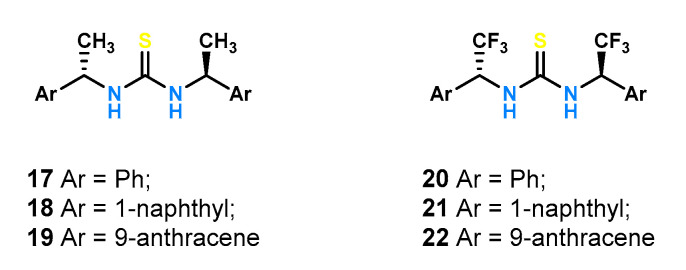
Simple symmetric thiourea receptors **17**–**22**.

To investigate the effect of steric hindrance, receptors **17**–**19** (Figure 8) have been synthetized [38]. The thiourea function was linked to a phenyl, 1-naphthyl, and 9-anthracene substituent, respectively, via a methine bridge. Additionally, for each receptor, its counterpart with a trifluoromethyl instead of a methyl group was obtained by synthesizing receptors **20**–**22** (Figure 8). Ligands dedicated to binding mandelates were evaluated using ^1^H NMR titrations in a competitive solvent such as DMSO-*d_6_*.

Replacement of the methyl with a trifluoromethyl group (both of which create nearly identical steric hindrance) resulted in a tenfold increase in the stability constants of the resulting complexes with the highest K*_R_* = 447 M^−1^ and K*_S_* = 205 M^−1^ for the *R*- and *S*-enantiomers, respectively, and consequently the best chiral recognition (K*_S_*/K*_R_* = 2.18) recorded for the naphthyl substituted receptor **21** (compared to its counterpart with a methyl group **18**: K*_R_* = 14 M^−1^ and K*_S_* = 18 M^−1^ and K*_S_*/K*_R_* = 1.29). Replacement of the naphthyl substituent by phenyl and anthracene resulted in the reduction of both binding affinity and enantioselectivity for mandelate, K*_S_*/K*_R_* = 1.64 and K*_S_*/K*_R_* = 1.38 for **20** and **22**. These results highlight that even a small structural change in the receptor framework has a large effect on the chiral recognition properties.

**Figure 9:**
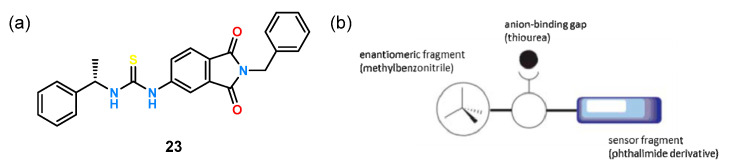
(**a**) The structure of receptor **23** and (**b**) its mode of operation as a chemosensor.

An example of an asymmetric thiourea receptor designed to act as a chiral chemosensor is ligand **23**, shown in Figure 9a [39].

The thiourea group responsible for anion binding was combined on one side with a phthalimide derivative to serve as the sensing function and on the other side with a chiral benzyl derivative to serve as the enantioselective fragment (Figure 9b). When evaluating the enantioselective properties of ligand **23** by UV-Vis titration experiments in acetonitrile, little change in absorption was observed when anions of the corresponding lactic acid enantiomers were added. Only the comparison of the change in absorption with the inverse of the guest concentration showed clear differences for the complexes formed and allowed the calculation of the corresponding stability constants for both guest enantiomers and the determination of K*_D_*/K*_L_* = 1.93.

**Figure 10:**
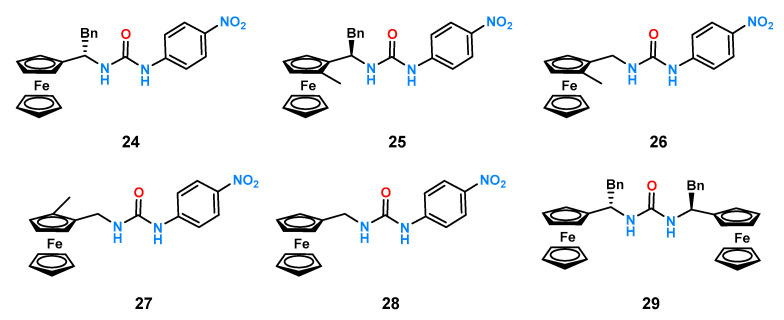
Chiral urea receptors **24**–**29** containing ferrocene unit.

Another example of smartly designed ligands is the family of chiral, electrochemically functional receptors developed by Tucker’s group [40,41]. They investigated a series of ligands containing in their structure a (thio)urea unit as an anion binding center to which nitrobenzene was attached on one side (the presence of which made UV studies possible), while on the other, a ferrocene derivative was attached via a methine bridge as an electrochemically active unit. The effect of steric hindrance on chiral recognition was determined using UV-Vis titration experiments in acetonitrile. Evaluation of receptors **24**–**29** with a set of selected chiral anions was performed (Figure 10).

**Figure 11:**
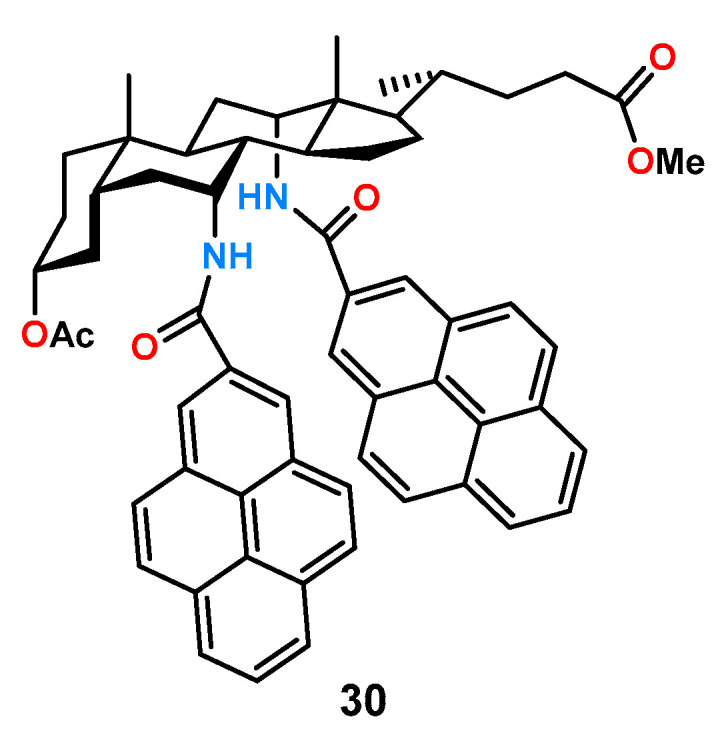
Receptor **30** based on cholic acid decorated with two pyrene fluorophores.

Cholic acid, due to its easy availability and inherent chirality, is attractive as a building block of chiral receptors for anion recognition. An example of such a ligand is the chiral receptor **30** (Figure 11) which is also a fluorescent sensor due to the presence of two pyrene substituents [21].

Studies on the potential enantioselectivity of ligand **30** were carried out by fluorescence titration experiments in acetonitrile with a pair of enantiomers of mandelic acid. The results show practically unnoticeable changes in fluorescence intensity for the *R*-enantiomer, while for the *S*-enantiomer, the change was significant and allowed the determination of the stability constant of the complex as K*_S_* = $3.43\times 10^{3}\;M^{-1}$. Chiral recognition of mandelic acid anions was estimated as K*_S_*/K*_R_* = 5.0.

**Figure 12:**
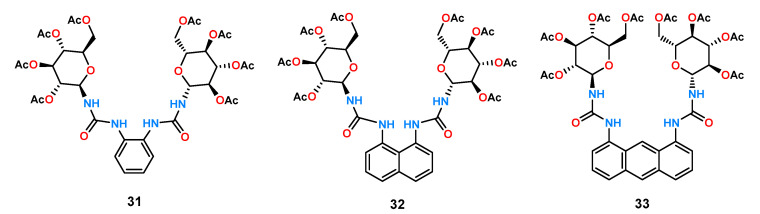
Urea-glucopyranose receptors **31**–**33**.

An example of a study of the effect of binding pocket size on anion association and chiral recognition by a family of three symmetrical urea-glucopyranose derivatives **31**–**33** was published by the Jurczak group (Figure 12) [42].

The receptors tested differed in geometry and binding pocket size, with naphthalene receptor **32** having the smallest gap, benzene receptor **31** having a slightly larger, and anthracene receptor **33** having the largest one. Preliminary titration studies of the model carboxylates (acetate and benzoate), performed under ^1^H NMR control in DMSO-*d_6_* containing 0.5% of water H_2_O, showed a stronger affinity for the smaller and more basic acetic anion for all three receptors. The stability constants of the complexes of both anions (K*_AcO_*/K*_BzO_*) compared with each other determine the dependence of the size of the binding pocket on the ratio of these constants. It turned out that, for receptor **33,** this ratio was 10, while for the other two, it was 5 and 3 for **32** and **31**, respectively. Then, using the same technique, the affinity of the obtained receptors for the anions of mandelic acid and tryptophan derivatives (*N*-Boc-Trp) was examined. The two receptors **31** and **32**, which have smaller binding pockets, were characterized by low chiral anion recognitions (1.05 and 0.88 for **31** and 1.07 and 1.12 for 32 for mandelate and the protected amino acid, respectively). On the other hand, for receptor **33**, relatively good results of K*_D_*/K*_L_* = 1.25 and 1.81 were obtained. Subsequently, analogous studies of this receptor with *N*-Boc-Phe and *N*-Boc-Val were carried out, for which satisfactory results of the ratio of constants of complexes K*_D_*/K*_L_* = 2.42 and 2.07 were obtained for valine and phenylalanine, respectively.

**Figure 13:**
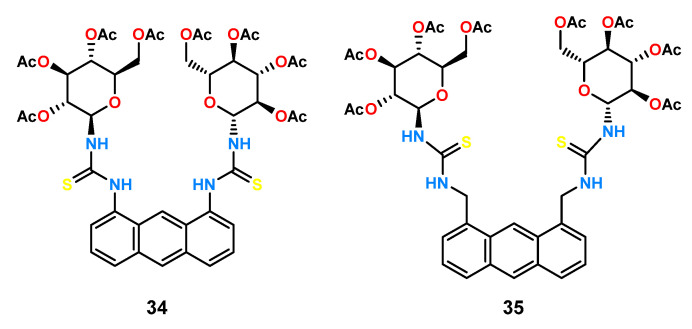
Anthracene receptors **34** and **35** decorated with thiourea glucopyranoses.

Analogous thiourea fluorescent chemosensors, using the anthracene platform, were published by Kim and coworkers [43]. The receptors **34** and **35** (Figure 13) were synthetized, and their chiral recognition for two sets of anions, *N*-Boc and DBN-protected tetrabutylammonium salts of α-amino acid—alanine, valine, threonine, leucine, phenylglycine, and phenylalanine—was measured by the UV-Vis titration experiments in MeCN.

The addition of amino acid derivatives to both **34** and **35** receptor solutions resulted in a decrease in fluorescence intensity due to a chelation-enhanced quenching (CHEQ) reaction. For receptor **34**, the CHEQ effect was explained by photoinduced charge transfer (PCT), while the CHEQ effect for receptor **35** was explained by photoinduced electron transfer (PET). Comparison of a series of results obtained for *N*-blocked amino acids to anions having DNB as a protecting group showed that, in the second case, the effect of lowering the fluorescence intensity is even greater. This is explained by the presence of nitro groups which usually further decrease the fluorescence of both PET and PCT. The fluorescence lowering effect for receptor **34**, with *d*- and *l*-*N*-Boc-phenylglycine was 22.5% and 26.1%, respectively, while for *d*- and *l*-*N*-DNB-phenylglycine, it was 66.6% and 63.9%. The obtained stability constants of the complexes were calculated using the out-dated Benesi–Hildebrandt approximation (vide infra). The best enantioselective binding result was obtained for *N*-Boc-phenylglycine. Receptor **34** bound the *d-* and *l-*enantiomers with stability constants of 2160 and 11,800 M^−1^, respectively, providing a ratio of K*_D_*/K*_L_* constants equal to 5.50. Different results were obtained for receptor **35**, for which the stability constant for the formation of a complex with the *d*-enantiomer was 2300 M^−1^, while for the *l*-enantiomer, it was 23,900 M^−1^. The ratio of the K*_L_*/K*_D_* constants was 10.4. This unexpected relationship was explained by the formation of a CH-π interaction between the anthracene backbone and the methylene group in the receptor-**35**–alanine complex. This type of interaction was impossible for **34** which lacks methylene groups in its structure, potentially responsible for the conformational lability of the entire system. However, a closer look at the Supporting Information indicates that the Korean authors misinterpreted the data. Jurczak and coworkers [16,44] questioned the results obtained by the Koreans by performing a competent ^1^H NMR titration of receptor **34** with *N*-Boc-Ala and *N*-Boc-Val. The determined chiral recognition in this case was 1.31 and 1.22 compared to the 5.50 and 4.30 presented in the paper for alanine and valine, respectively. The obtained ratios of stability constants were confirmed by fluorometric titration. The results obtained by Kim’s group are subject to a powerful error due to the use of the Benesi–Hildebrandt approximation because of the complicated fluorescence quenching mechanism.

**Figure 14:**
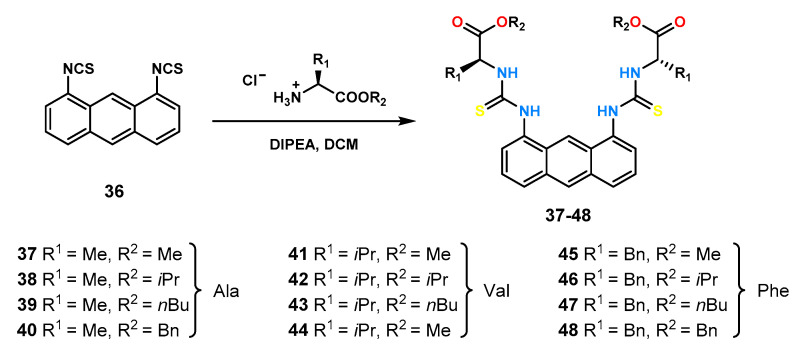
A family of 1,10-Dithiourea anthracene receptors **37**–**48** decorated with α-amino acids.

1,10-Dithiourea anthracene derivative was used to obtain a static library of thiourea receptors containing derivatives of different amino acids [16]. Twelve new ligands (Figure 14) were obtained by reacting dithioisocyanate **36** with ester derivatives of three amino acids (alanine, valine, and phenylalanine). Four esters of various sizes and shapes (methyl, isopropyl, *n*-butyl, and benzyl) were used to investigate the effect of ester group size on the stability constant value and chiral recognition.

**Figure 15:**
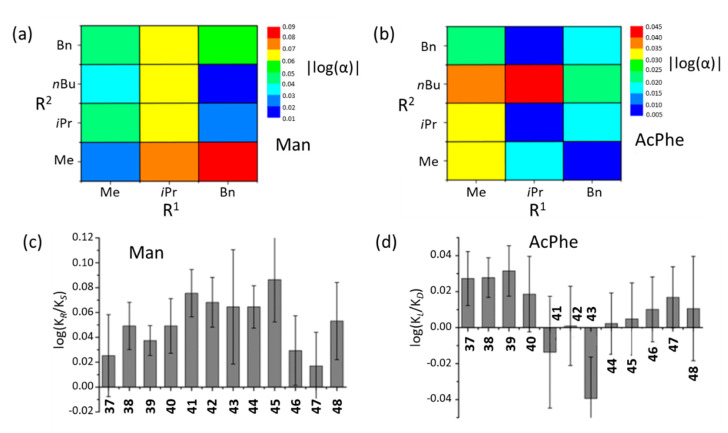
(**a**,**b**) Enantioselectivity distribution (log(α)) of receptors with mandelates and acetylated phenylalanines; (**c**,**d**) graphical analysis of the effect of R^1^ and R^2^ groups on the enantioselectivity of receptors with mandelates and acetylated phenylalanines.

The ability to enantioselectively bind carboxylates was examined by competent titration under the control of ^1^H NMR in acetonitrile-*d_3_* with model pairs of enantiomers of mandelic acid and *N*-Ac-Phe anions [45]. A comparison was made by counting K′ = K/K (R^1^ = Me) for the constant R^2^ and the given anion. The highest constants were obtained for valine derivative receptors **41**–**44** (K′ = 2.33–2.91), despite having the largest isopropyl substituent by volume (according to Taft steric parameters) [46,47], while phenylalanine derivatives showed medium affinity (K′ = 0.50–0.76) (Figure 15a).

An analogous comparison was made for the substituent of the ester group assuming K′ = K/K (R^2^ = Me). In this case, the substituent effect (*i*Pr < *n*Bu < Bn ≈ Me) was much smaller (Figure 15b). All receptors tested showed a higher affinity for the *R-*enantiomer of mandelate (Figure 15c), whereas no such selectivity was present for the phenylalanine enantiomers (Figure 15d). The obtained K*_R_*/K*_S_* ratios of the studied receptors were in the range of 1.0–1.22 which qualifies for quite low chiral recognition values. The described studies show a significant effect of steric substituents on the height of the persistence constant of the complexes.

**Figure 16:**
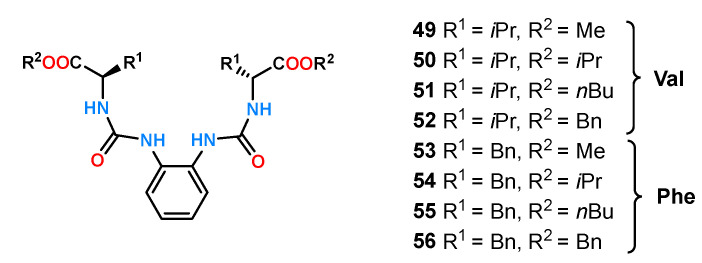
A library of 1,2-dimethylbenzene receptors **49**–**56**.

A similar analysis was performed for a family of 1,2-diurea benzene ligands [15]. Using the UV-Vis titration experiments in MeCN, two groups of receptors derived from valine and phenylalanine with different substituents in the ester group (**49**–**56**, Figure 16) were compared.

Very similar results were obtained as for the anthracene receptors described above. The enantioselectivity of the described receptors was low, and there was a strong dependence of the values of stability constants on the size of the substituents.

**Figure 17:**
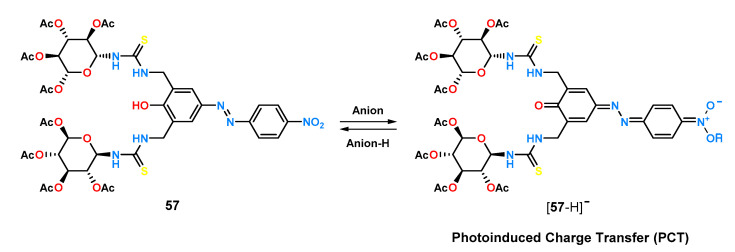
Chromophore receptor **57.**

An example of a receptor with an intentionally introduced chromophore fragment that was designed for chiral recognition is compound **57** [48]. In its structure, it contains thiourea groups responsible for carboxylate ion binding, glucopyranose fragments responsible for chirality, and an azophenol system as a chromophore (Figure 17).

Receptor **57** exhibits pronounced colorimetric changes upon addition of anions which can be attributed to the anion-induced deprotonation of an azophenol moiety allowing the occurrence of a photoinduced charge transfer (PTC). This property was employed to study the chiral recognition properties of **57** using the UV-Vis titration technique in acetonitrile. Ligand **57** showed good enantioselectivity for anions derived from α-amino acids protected by a *tert*-butoxycarbonyl (Boc) group, with a preference for *D*-amino acids and a chiral recognition for alanine of 3.60 (Table 2). For the anions of the same amino acids, protected by a dinitrobenzyl group (DNB), the enantioselectivity (K*_D_*/K*_L_*) was determined to be medium, ranking between 1.10 and 2.55. For sensor **57**, tests were also carried out with the chiral salts of naproxen and 2-phenylpropionic acid, for which the ratio of the constants of the K*_R_*/K*_S_* complexes was 1.86 and 2.95, respectively.

**Figure 18:**
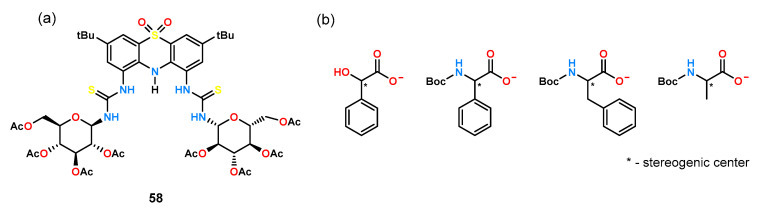
(**a**) Phenothiazine diurea receptor **58** and (**b**) evaluated hydroxy and amino acids.

Huszthy’s group [49], which has conducted extensive research on the fundamentals of chiral cation and anion recognition phenomena and their applications, has developed several diurea receptors with *C2* symmetry. Among others, they used a rigid platform of 5,5-dioxophenothiazine, whose diamine derivative was reacted with per-*O*-acetylated glucosamine isocyanate to obtain receptor **58** (Figure 18).

The ligand thus synthesized was tested for enantioselective anion recognition using UV-Vis titration experiments in MeCN, using TBA salts of acids with a stereogenic center on the α carbon atom (Figure 18b, Table 3). The studies showed that groups located at the stereogenic center have a strong effect on enantioselectivity. For anions having a phenyl group attached to the α carbon atom (mandelate, phenylglycine), chiral recognition was moderate. When the aromatic ring was offset from the stereogenic center by a methylene group (phenylalanine), negligible enantioselectivity was observed. In contrast, when the aromatic substituent was replaced with an aliphatic one (alanine), no enantiomeric differentiation by the receptor was observed at all. It was also observed that shifting the phenyl ring away from the carboxyl group by a methylene group reduces the interaction between the anion and the receptor. The effect of the anion protecting group on chiral recognition was also tested by measuring stability constants for amino acids protected by a formyl (Form), acetyl (Ac), *tert*-butylxycarbonyl (Boc), and pivaloyl (Piv) group. Receptor **58** showed the best chiral recognition for amino acids protected with Boc and Piv; for amino acids protected with a formyl group, the effect on enantioselectivity was negligible.

**Figure 19:**
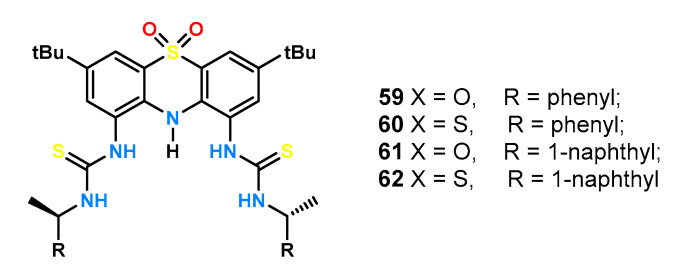
Phenothiazine receptors **59**–**62**.

The same phenothiazine derivative was then used to obtain a series of ligands **59**–**62** (Figure 19) and to investigate their stereo-differentiating properties [50]. The effects of the acidity of the hydrogen bond donors and the size of the aromatic substituent linked to the thio(urea) function via a methine bridge were examined.

An identical set of enantiomeric amino acid pairs was tested as for the glucopyrazone derivative (Figure 18b). Urea derivatives **59** and **61** exhibited lower absorption and smaller fluorescence changes compared to thiourea ligands **60** and **62** making it impossible to determine reliable stability constants of the complexes by titrations under UV-Vis control in MeCN. Both, receptor **59**, containing a benzyl unit in its structure, and receptor **61**, being a naphthyl derivative, showed little enantioselectivity, with the receptor bound to the larger aromatic substituent, showing slightly higher differences, e.g., for *N*-Boc-Phg ΔlogK = 0.24 compared to ΔlogK = 0.03 for **59** and **61**, respectively. The results presented here suggest that chiral recognition depends on very subtle effects.

**Figure 20:**
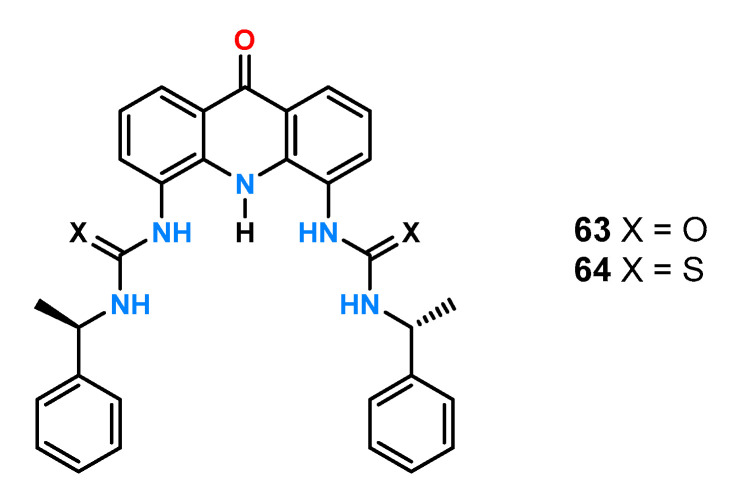
Receptors **63** and **64** using the acridinium-9-one platform.

Huszthy and coworkers [51] also obtained two receptors (urea **63** and thiourea **64**, Figure 20) based on the acridinium-9-one platform.

Results from the UV-Vis titration experiments in MeCN containing 1% DMSO using reference anions with a stereogenic center on the α-carbon atom (Figure 18b) showed a particularly high degree of chiral recognition for *N*-Boc-Ala by the urea receptor **63** (ΔlogK = −0.56; α = 3.6), compared to anions having an aromatic substituent (Table 4). Furthermore, changing the urea group to thiourea had a significant effect on the chiral recognition of phenylglycine, with ΔlogK = 0.08; α = 1.2 for receptor **63** and ΔlogK = 0.26; α = 1.8 for ligand **64**.

**Figure 21:**
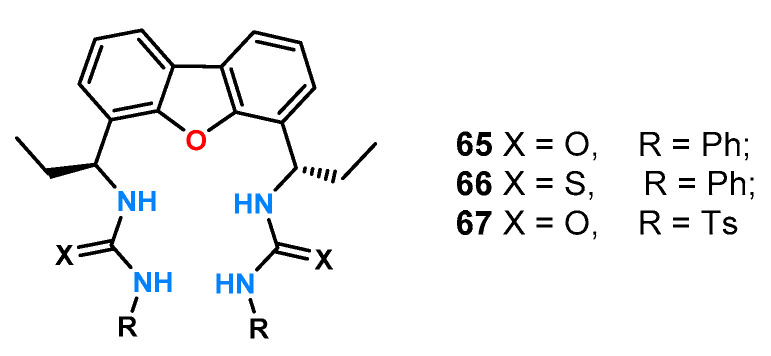
Dibenzofuran receptors **65**–**67.**

A few years ago, a study on the enantioselectivity of symmetric diurea receptors forming host:guest complexes with a stoichiometry of 1:2 was published [52]. The authors obtained three receptors **65**–**67** (Figure 21), using a dibenzofuran derivative as the structural backbone of the receptors, and placed a source of chirality between the platform and hydrogen bonding donors. They decided to investigate their ability to enantioselectively bind carboxylate anions by titration experiments under ^1^H NMR control while planning to use them as chemical shift agents (CSAs).

**Scheme 1:**
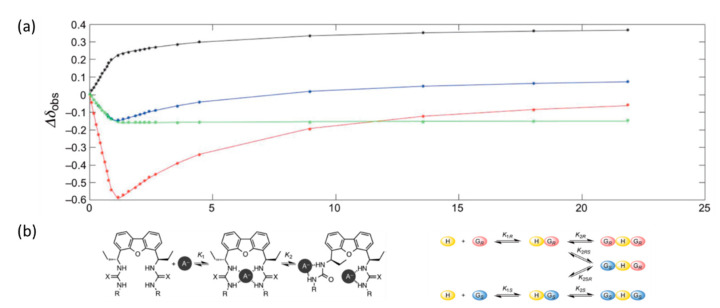
(**a**) Characteristic movement of signals on NMR spectrum and plot of shift change against number of receptor equivalents, and (**b**) proposed anion binding mechanism.

The one from the hydrogen atom attached to the α carbon atom of the mandelic acid anion was chosen as the reference signal in this study. In preliminary studies, the effect of solvent on the potential for enantioselective recognition by receptor **65** was tested by conducting titrations of a racemic mixture of *DL*-tetrabutylammonium mandelate (TBAM) in three solvents: CDCl_3_, DMSO-*d_6_*, and acetone-*d_6_*. The largest separation of the enantiomeric signals after the addition of two equivalents of guest anions was obtained for chloroform ΔΔδ = 0.051 ppm, followed by acetone ΔΔδ = 0.042 ppm, and the smallest for DMSO ΔΔδ = 0.026ppm. Moreover, the best separation of the traced signal was achieved for 0.5 eq of the mixture in acetone (ΔΔδ = 0.063 ppm). The characteristic shifts of the signals in the ^1^H NMR spectrum (Scheme 1a) during titration are indicative of conformational changes of the ligand and the formation of a complex with complex stoichiometry, in this case, mixed 1:1 and 1:2. The binding model proposed by the authors is shown in Scheme 1b.

Receptors **66** and **67** also showed enantioselectivity toward the racemic mixture of *DL*-TBAM. The thiourea analog **66** behaved comparably to ligand **65**, but a smaller chemical shift difference of ΔΔδ = 0.041 ppm was observed for the two equivalents. For the tosyl derivative, the value of ΔΔδ increased continuously with the addition of the receptor, reaching ΔΔδ = 0.53 ppm, suggesting the formation of a complex with a host:guest stoichiometry of 1:1. For the best receptor **65** in this combination, the authors tested its effect on a number of mixtures of racemic α-hydroxy and α-amino acid derivatives.

**Figure 22:**
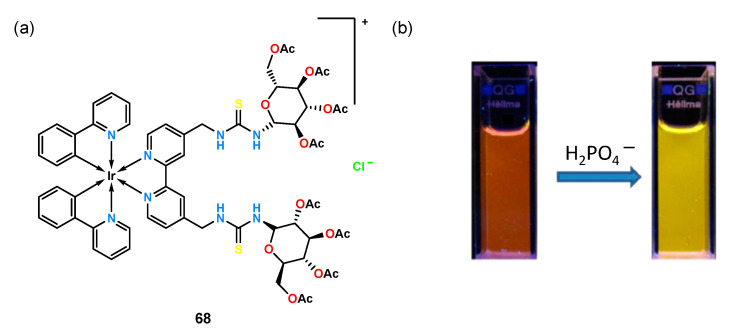
(**a**) Iridium receptor **68** and (**b**) phosphorescence changes by naked eye upon the addition of TBA-H_2_PO_4_ to CH_3_CN.

An approach combining the functions of a phosphorescent sensor with a preorganized carboxylate ion binding pocket and a chiral fragment was presented by Yoon and coworkers [53]. They used an amine derivative of the iridium(III) complex as a rigid platform which they reacted with a thioisocyanate derivative of per-*O*-acetylated glucosamine to obtain receptor **68** (Figure 22).

Experiments for chiral recognition were performed by titration under UV-Vis and phosphorescence in acetonitrile with two sets of *N*-protected amino acids: alanine, valine, threonine, leucine, phenylglycine, and phenylalanine. Receptor **68** exhibited high association constants of the resulting complexes and had a higher affinity for the *D*-enantiomers when analyzing amino acids protected with a *tert-*butoxycarbonyl (Boc) group, with the best chiral recognition obtained for leucine (K*_D_* = $1.1\times 10^{5}{\;M}^{-1}$, K*_L_* = $4.71\times 10^{4}{\;M}^{-1}$; K*_D_*/K*_L_* = 3.16). When ligand **68** was tested with a group of amino acids protected by a 3,5-dinitrobenzyl group, a greater phosphorescence quenching effect and a higher affinity for the *L*-isomers of the tested anions were observed compared to the previously tested set of amino acids. Receptor **68** showed the highest enantioselectivity for the threonine enantiomer pair equal to K*_L_*/K*_D_* = 3.04 (K*_D_* = $5.07\times 10^{3}{\;M}^{-1}$, K*_L_* = $1.54\times 10^{4}{\;M}^{-1}$).

**Figure 23:**
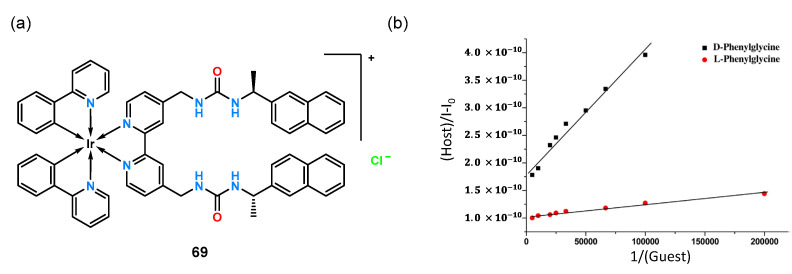
(**a**) Diurea receptor **69** containing iridium complex and (**b**) linear dependence of the phosphorescence intensity to the inverse of the guest concentration.

Using the same iridium platform, a diurea receptor **69** (Figure 23a) was constructed with a chiral 1-naphthylethyl fragment attached [54].

Analysis for chiral recognition was performed by UV-Vis titration experiments in MeCN, using, as before, the phorescence properties of the iridium(III) complex. The ligand tested exhibited extremely high *N*-Boc-Phg enantioselectivity, binding the enantiomeric pair at a ratio of K*_D_*/K*_L_* = 5.0. Figure 23b shows the linear relationship of the Benasi–Hildebrand equation of phosphorescence intensity to the inverse of the guest concentration which demonstrates the high value of chiral recognition for this anion.

**Figure 24:**
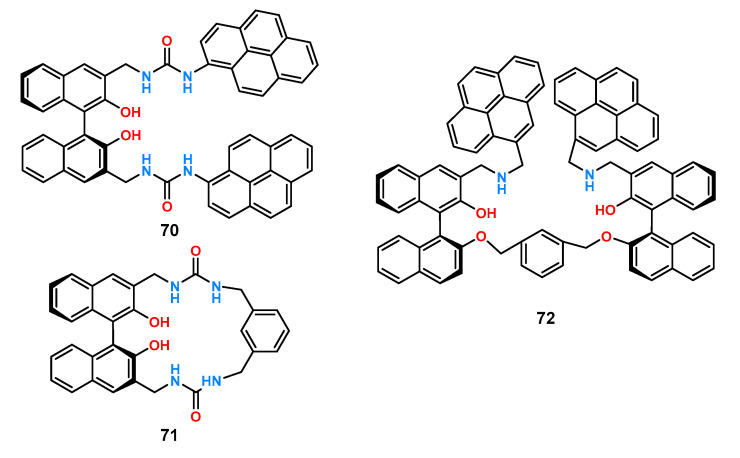
Family of receptors **70**–**72** containing BINOL moiety**.**

A representative group of chiral anion receptors having BINOL in their structure is ligands **70**–**72** (Figure 24) [55]. Two urea groups were attached to the rigid platform to form a suitable pocket for binding carboxylate anions and, in addition, pyrene functions responsible for the fluorescence response (receptors **70** and **72**).

Compounds **70**–**72** were screened for chiral recognition with a set of tetrabutylammonium salts of amino acids protected by a *tert*-butoxycarbonyl group (Phe, Leu, Ala, Ser) by fluorescence titration experiments in DMSO. All ligands exhibited higher affinity toward the *d*-enantiomers. Receptor **70** showed the highest chiral recognition for the alanine derivative with fluorescence quenching ΔI*_D_*/ΔI*_L_* = 6.1 which was also confirmed by ^1^H NMR spectra by observing and comparing the N-H shifts of protons for both enantiomers (Δδ = 0.154 and Δδ = 0.139, for *d*- and *l*-alanine, respectively). Compared to the linear receptors **70** and **72**, whose fluorescence intensity decreased in each case, the addition of an anion to the macrocyclic host **71** resulted in a different behavior, namely, a signal enhancement at 258 nm, a decrease at 342 nm, and an enhancement at 370 nm. The highest enantioselectivity was obtained for receptor **72** using the enantiomeric pair of alanine derivatives ΔI*_D_*/ΔI*_L_* = 12.95.

**Figure 25:**
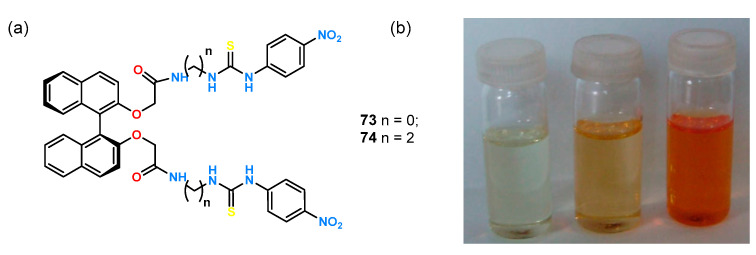
(**a**) BINOL receptors **73** and **74**; (**b**) effect of the anion addition on color changes of receptor **74** in DMSO. From left to right, **74** (5.0×10−5 molL), **74** (5.0×10−5 molL) + 30 eq of *R*-mandelate, **74** (5.0×10−5 molL) + 30 eq of *S*-mandelate.

BINOL was also used to design colorimetric receptors **73** and **74** (Figure 25) [56]. Thiourea functions and *p*-nitrophenyl groups were symmetrically attached to the chiral rigid platform. The receptors differed in the distance of the binding pocket from the chiral fragment by two methylene groups.

Preliminary enantioselectivity studies for both hosts were performed by fluorescence absorption experiments in DMSO for a pair of mandelates. While the addition of the enantiomeric guest to receptor **73** resulted in nearly identical fluorescence quenching for both isomers (from which it can be inferred that the ligand exhibits low enantioselectivity), the quenching efficiency for receptor **74** was equal to ΔI*_D_*/ΔI*_L_* = 3.3. Experiments leading to the determination of stability constants and ΔI*_D_*/ΔI*_L_* for anionic derivatives of malic acid and phenylglycine were also performed, but both **73** and **74** showed no chiral recognition toward the tested guests. To further evaluate receptor **74** association of a pair of enantiomeric mandelic acids, titrations were carried out under ^1^H NMR control in DMSO-*d_6_* for the racemic mixture and the single isomers. Changes in the downfield shift of thiourea protons were observed, with maximum shifts of δ = 10.23 and δ = 9.20 ppm upon addition of *d*-enantiomer, and 10.97 and δ = 9.39 when *l*-enantiomer was added. The resulting enantioselectivity was explained by the presence of an additional π-π interaction of receptor **74** with *d*-mandelate, the presence of which was confirmed by molecular modeling.

**Figure 26:**
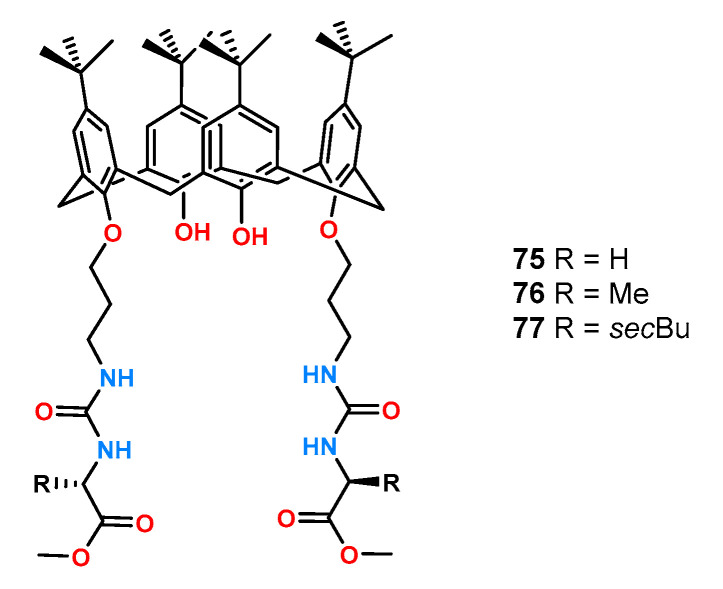
*C*_2_-Symmetrical calixarene diurea receptors **75**–**77**.

Receptors **75**–**77** based on the calixarene (Figure 26) were formed from the symmetric attachment of urea functions to the lower rim of calixarene and were examined by ^1^H NMR titration experiments in CDCl_3_ and acetone-*d_6_* [57].

Surprisingly, all ligands formed complexes with higher stability constants in acetone compared to chloroform, which is a less competent solvent medium. This is attributed to the strong intramolecular hydrogen bonds which were broken in a more polar solvent such as acetone, allowing the formation of complexes with the anion. The highest enantioselectivity toward the tested pair of phenylalanine enantiomers was characterized by receptor **77** (K*_D_*/K*_L_* = 4.14), compared to K*_D_*/K*_L_* = 3.55 and K*_D_*/K*_L_* = 1.96 for **76** and **75**, respectively.

**Figure 27:**
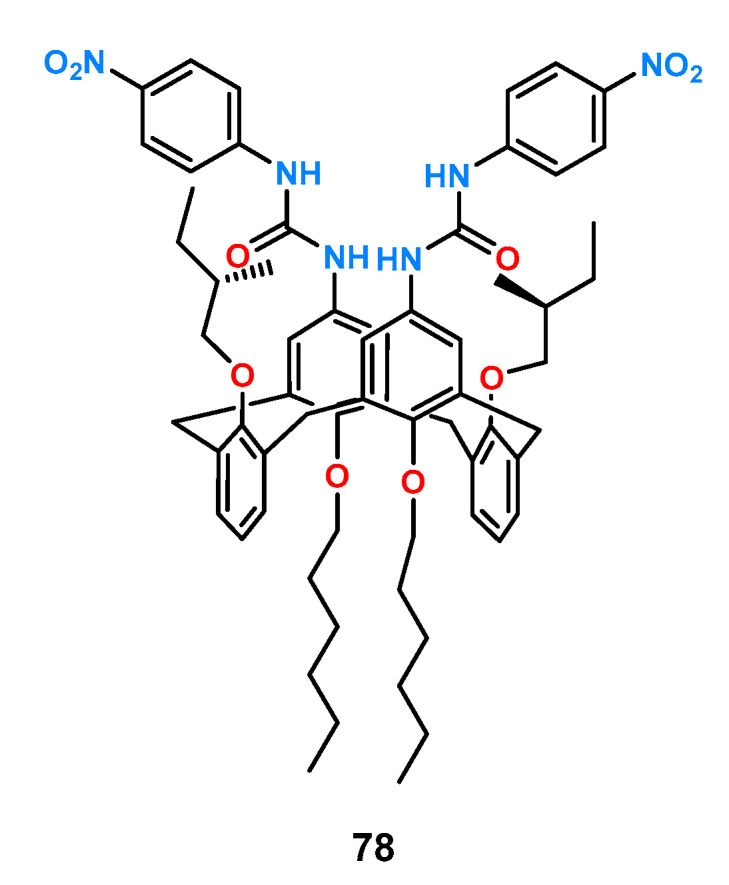
C_2_-Symmetrical calixarene diurea receptor **78.**

In the case of receptor **78**, urea functions were attached to the core ring of calixarene (Figure 27) [58].

Its binding properties were examined by ^1^H NMR titration experiments in DMSO-*d_6_*. When this host was evaluated with a number of chiral carboxylates (Phe, *N*-Ac-Phe, *N*-Ac-Leu, *N*-Ac-Trp, and Man), it was shown that **78** did not show significant enantioselectivity toward the protected amino acids, whereas for free phenylalanine, the ratio of association constants for both stereoisomers was K*_D_*/K*_L_* = 2.86.

## 4. Hybrid Receptors

**Figure 28:**
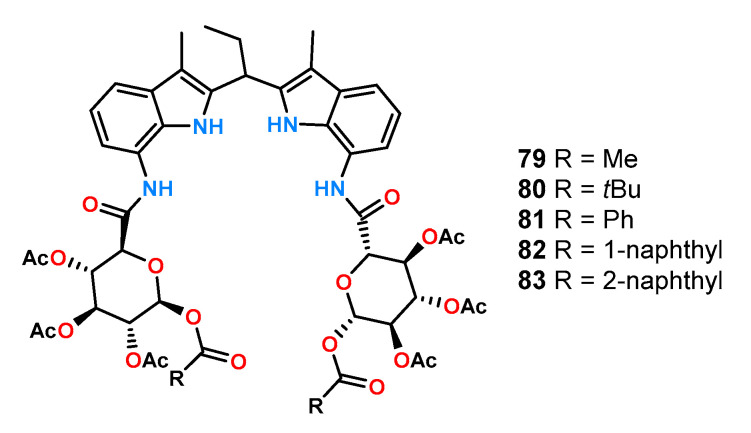
Sugar receptors **79**–**83**.

Among the effective receptors for chiral carboxylates, there are also ligands constructed with simultaneous insertion of hydrogen bonding donors of different nature into the receptor structure. A good representative example is the formation of a hybrid of two indole molecules via a methine bridge together with two amide functions which creates an anion binding pocket. Using this 7,7′-Diamino-2,2′-diindolylmethane – (DIM)-based hybrid concept, a series of chiral receptors **79**–**83** was synthetized in the Jurczak group (Figure 28) [59].

These receptors were tested for their ability to recognize anions by the ^1^H NMR titration technique in a highly competitive solvent, such as DMSO-*d_6_* with 0.5% H_2_O (*v*/*v*). The receptors differed in the protecting group of the hydroxyl group on the anomeric carbon atom. The results of chiral recognition of a range of anions using the receptors described above are given in Table 5.

These results show very high ratios of association constants, which indicates an excellent matching of the bonding gap to the studied anions and appropriate localization of the chiral part. Moreover, a significant increase in chiral recognition was observed for receptors possessing an aromatic substituent which may indicate the presence of additional π-π interactions.

**Figure 29:**
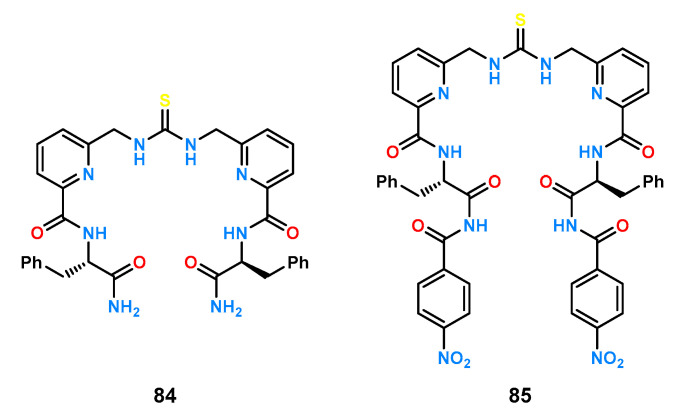
Hybrid receptors **84** and **85.**

Another example of hybrid receptors is the acyclic ligands **84** and **85** with a central thiourea function (Figure 29) [60].

Titrations under ^1^H NMR control in a mixture of 10% DMSO-*d_6_* in CDCl_3_ (*v*/*v*) of a series of protected amino acids (Ala, Phe, Asn, Gln, Trp, Ser) showed that receptor **84** exhibited little enantioselective amino acid binding properties. However, it exhibited high selectivity toward individual guest pairs; for example, the complex formed between **84** and *N*-Ac-Trp had 30 times higher stability than the **84** and *N*-Ac-Ser complex. In contrast, host **85** showed chiral recognition for the enantiomeric pair of *N*-acetylated tryptophan derivatives (K*_L_* = 1925, K*_D_* = 3785 M^−1^).

**Figure 30:**
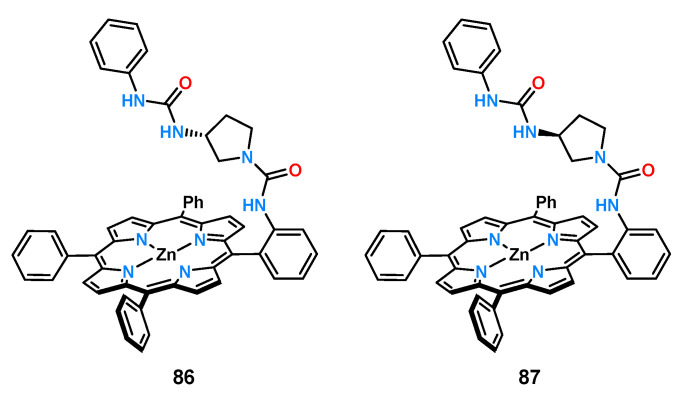
Porphyrin receptors **86** and **87.**

An interesting example demonstrating how small changes in receptor structure can affect chiral recognition are receptors **86** and **87** which differ in the absolute configuration of the carbon atom of the chiral fragment (Figure 30) [61].

**Scheme 2:**
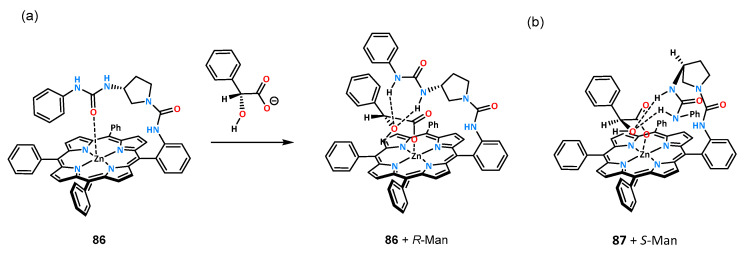
Binding mode of receptor **86** with *R*-mandelate (**a**) and receptor **87** with *S*-mandelate (**b**).

The binding properties of ligands constructed on a rigid porphyrin platform toward an enantiomeric pair of mandelic acid anions were investigated by titrations under UV-Vis and ^1^H NMR control in chloroform. The slight difference between the receptors resulted in a reversal of chiral recognition for mandelates. Receptor **86** showed a higher affinity for the *R*-stereoisomer, with K*_R_*/K*_S_* = 3.0, while host **87** bound the *S*-enantiomer with a higher association constant of K*_R_*/K*_S_* = 0.32. The authors also proposed a binding model for both receptors which is shown in Scheme 2.

The oxygen atom of the urea carbonyl group for receptors in the free state (no guest addition) coordinates to the zinc atom located in the porphyrin system, and upon anion addition, the receptor begins to preorganize. The anion molecule interacts with the metal center on one side and with the urea function through hydrogen bonds on the other side. Ligand **86** may interact more favorably with *R*-mandelate due to its better fitting to the porphyrin site, and there is no steric interaction with the phenyl group; however, if the stereoisomer *S* binds in a similar manner, there will be an interaction of the phenyl substituent with the porphyrin surface.

**Figure 31:**
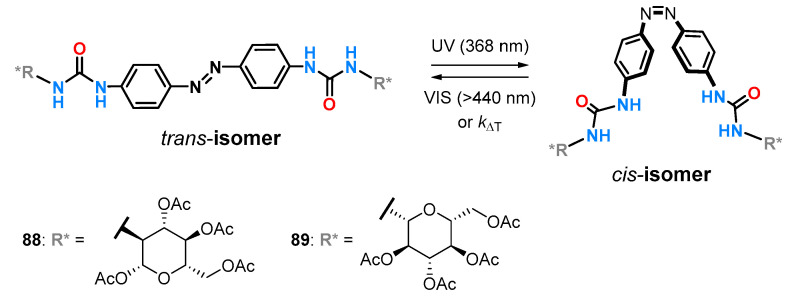
Photoswitchable symmetrical sugar-decorated urea-azobenzene receptors **88** and **89.**

The symmetrical molecular receptors **88** and **89** consisting of a photoswitchable azobenzene scaffold functionalized with urea hydrogen-bonding groups and *d*-carbohydrates as chiral selectors were developed by Dąbrowa et al. to gain control over the chiral recognition of α-amino acid derived carboxylates (Figure 31) [18].

Upon irradiation with the UV light, the native and planar *trans*-isomers of receptors **88** and **89** readily undergo *trans*→*cis* isomerization to the corresponding metastable and more compact *cis*-isomers. The reverse *cis*→*trans* isomerization is triggered upon photoirradiation with visible light or upon heating. The *trans* and *cis* isomers were found to exhibit different affinities, selectivities, and binding modes toward tetrabutylammonium salts of amino acids protected by a *tert*-butoxycarbonyl group (Boc-Phe and Boc-Trp) as revealed from UV-Vis and ^1^H NMR titration experiments in highly polar DMSO/H_2_O (99.5/0.5 *v*/*v*) solvent mixture (Table 6).

Specifically, the binding affinity for the same enantiomerically pure guest was up to 3-fold higher for *trans*-isomers than for *cis*-isomers of receptors **88** and **89**. Furthermore, both *trans*- and *cis*-isomers of **88** and **89** exhibited higher affinity to Boc-Trp over Boc-Phe, with a slight preference for the carboxylate guests derived from *D*-amino acids (except *trans-***88**, which binds *L*-Boc-Phe more strongly than *D*-Boc-Phe). The preference for the Trp anion was attributed to a favorable intermolecular interaction between anion and receptor, in particular between indole NH proton and the carbonyl group of the sugar moieties. Moreover, the rate of thermal *cis*→*trans* isomerization was found to depend on the chiral binding ability properties of the *cis*-isomer; that is, a more strongly bound carboxylate enantiomer, as well as a higher enantiomer concentration, caused faster relaxation to the corresponding *trans*-isomer of receptors **88** and **89**.

## 5. Conclusions

Designing electrically neutral chiral receptors for anions is a very challenging task. There is no doubt about the tremendous importance of chirality in biological systems and the ubiquity of chiral molecules containing carboxylic acid (that is deprotonated under physiological conditions). During the design of suitable chiral and enantioselective receptors and subsequent evaluation of their anion binding properties, many factors must be taken into account, such as the model of anion–receptor interaction, solvation energy, the possibility of the formation of complex systems, the influence of the solvent medium on the resulting diastereoisomeric pairs, or finally, the appropriate design of the geometry of the anion binding pocket and the chiral fragment.

First of all, we can distinguish two main strategies for composing such ligands. In the first, a well-matched carboxylate anion binding pocket is first developed, and then chiral fragments are attached to it; in the second, the binding pocket is created around the inherently chiral molecule. Both concepts work well in terms of developing effective receptors. A typical receptor has a binding pocket built of appropriate hydrogen bonding donor motifs and a chiral fragment responsible for chirality and is intended to perform specific functions. For example, a fluorescence or colorimetric sensor can be designed by the attachment of pyrene and nitrobenzene units, respectively. Likewise, an electrochemical response can be achieved by the incorporation of the redox-active ferrocene unit. Finally, chiral recognition in more demanding organic-solvent–water mixtures can be accomplished by receptors having the appropriately planned geometry of the binding pocket.

The role played by individual hydrogen bonding motifs is substantial. Their number, distribution, and acidity will determine how effective the ligand will be in a given solvent. As we saw in the examples shown above, a small modification is enough to change the enantioselective properties of the host.

## Figures and Tables

**Table 1:** Association constants and chiral recognition K*_D_*/K*_L_* for receptors **6**, **7**, and **8** with TBA salts of *d*- and *l*-Phe, respectively, in DMSO.

Anion	Receptor 6		Receptor 7		Receptor 8	
	K (M^−1^)	K*_D_*/K*_L_*	K (M^−1^)	K*_D_*/K*_L_*	K (M^−1^)	K*_D_*/K*_L_*
*d-*Phe	$5.94\times 10^{3}$	3.19	$7.64\times 10^{3}$	2.55	$1.24\times 10^{3}$	4.98
*l-*Phe	$1.86\times 10^{3}$		$3.00\times 10^{3}$		$2.49\times 10^{3}$	

**Table 2:** Association constants of complexes for **57** with TBA salts of Boc-protected amino acids in acetonitrile (1:1 binding model).

Anion	K*_D_* (M^−1^)	K*_D_*/K*_L_*	K*_L_* (M^−1^)
Phenylglycine	$3.80\times 10^{4}$	1.16	$3.27\times 10^{4}$
Leucine	$2.98\times 10^{4}$	2.22	$1.34\times 10^{4}$
Valine	$1.64\times 10^{4}$	1.43	$1.15\times 10^{4}$
Threonine	$6.89\times 10^{4}$	3.13	$2.20\times 10^{4}$
Alanine	$2.28\times 10^{3}$	3.60	$6.33\times 10^{2}$

**Table 3:** Stability constants of complexes for receptor **58** in acetonitrile.

Anion	logK	ΔlogK
*R-*Mandelate	4.92	0.19
*S-*Mandelate	4.73	
*D-*Boc-Phenylglycine	5.43	0.22
*L-*Boc-Phenylglycine	5.21	
*D-*Boc-Phenylalanine	5.88	0.09
*L-*Boc-Phenylalanine	5.79	
*D-*Boc-Alanine	5.94	0.04
*L-*Boc-Alanine	5.90	

**Table 4:** Association constants of complexes for receptors **63** and **64** in acetonitrile with 1% DMSO.

Anion	Receptor 63		Receptor 64	
	logK	ΔlogK	logK	ΔlogK
*R-*Mandelate	5.37	–0.01	5.52	0.20
*S-*Mandelate	5.38		5.32	
*D-*Boc-Phenylglycine	5.81	0.08	5.80	0.26
*L-*Boc-Phenylglycine	5.73		5.54	
*D-*Boc-Phenylalanine	6.38	0.05	6.43	0.06
*L-*Boc-Phenylalanine	6.33		6.37	
*D-*Boc-Alanine	6.08	–0.56	6.18	–0.25
*L-*Boc-Alanine	6.64		6.43	

**Table 5:** Chiral recognition of amino acids as tetrabutylammonium salts by receptors **79**–**83** in DMSO-*d_6_* + 5% H_2_O by titration under ^1^H NMR control.

Anion	Receptor									
	79	K*_R_*/K*_S_*	80	K*_R_*/K*_S_*	81	K*_R_*/K*_S_*	82	K*_R_*/K*_S_*	83	K*_R_*/K*_S_*
*S-*PhLac	259	3.68	328	2.41	250	3.44	317	4.14	304	3.46
*R-*PhLac	952		790		859		1312		1053	
*S-*Man	119	1.95	105	1.96	97	1.92	115	2.12	107	1.95
*R-*Man	233		205		187		245		209	
*D-*Boc-Trp	88	2.57	89	3.03	78	3.61	104	3.85	100	3.53
*L-*Boc-Trp	227		270		281		401		353	

**Table 6:** Chiral recognition of tetrabutylammonium salts of *N*-Boc-protected phenylalanine (Phe) and tryptophan (Trp) by *trans*- and *cis*-isomers of receptors **88** and **89** in DMSO-*d_6_* + 0.5% H_2_O by titration under ^1^H NMR control.

Anion	Receptor							
	*trans*-88	K*_D_*/K*_L_*	*cis*-88	K*_D_*/K*_L_*	*trans*-89	K*_D_*/K*_L_*	*cis*-89	K*_D_*/K*_L_*
*D-Boc-Phe*	55	0.62	30	1.00	63	1.19	39	1.25
*L-Boc-Phe*	89		30		53		31	
*D-Boc-Trp*	143	1.12	68	1.48	124	1.61	76	1.31
*L-Boc-Trp*	127		46		77		58	

## Data Availability

Data sharing not applicable.
